# PMC, a potent hydrophilic α-tocopherol derivative, inhibits NF-κB activation *via* PP2A but not IκBα-dependent signals in vascular smooth muscle cells

**DOI:** 10.1111/jcmm.12277

**Published:** 2014-04-13

**Authors:** Cheng-Ying Hsieh, George Hsiao, Ming-Jen Hsu, Yi-Hsuan Wang, Joen-Rong Sheu

**Affiliations:** a Department of Pharmacology, School of Medicine, Taipei Medical UniversityTaipei, Taiwan; b Graduate Institute of Medical Sciences, School of Medicine, Taipei Medical UniversityTaipei, Taiwan

**Keywords:** PMC, vascular inflammation, NF-κB, PP2A, ROS

## Abstract

The hydrophilic α-tocopherol derivative, 2,2,5,7,8-pentamethyl-6-hydroxychromane (PMC), is a promising alternative to vitamin E in clinical applications. Critical vascular inflammation leads to vascular dysfunction and vascular diseases, including atherosclerosis, hypertension and abdominal aortic aneurysms. In this study, we investigated the mechanisms of the inhibitory effects of PMC in vascular smooth muscle cells (VSMCs) exposed to pro-inflammatory stimuli, lipopolysaccharide (LPS) combined with interferon (IFN)-γ. Treatment of LPS/IFN-γ-stimulated VSMCs with PMC suppressed the expression of inducible nitric oxide synthase (iNOS) and matrix metalloproteinase-9 in a concentration-dependent manner. A reduction in LPS/IFN-γ-induced nuclear factor (NF)-κB activation was also observed in PMC-treated VSMCs. The translocation and phosphorylation of p65, protein phosphatase 2A (PP2A) inactivation and the formation of reactive oxygen species (ROS) were significantly inhibited by PMC in LPS/IFN-γ-activated VSMCs. However, neither IκBα degradation nor IκB kinase (IKK) or ribosomal s6 kinase-1 phosphorylation was affected by PMC under these conditions. Both treatments with okadaic acid, a PP2A-selective inhibitor, and transfection with PP2A siRNA markedly reversed the PMC-mediated inhibition of iNOS expression, NF-κB-promoter activity and p65 phosphorylation. Immunoprecipitation analysis of the cellular extracts of LPS/IFN-γ-stimulated VSMCs revealed that p65 colocalizes with PP2A. In addition, p65 phosphorylation and PP2A inactivation were induced in VSMCs by treatment with H_2_O_2_, but neither IκBα degradation nor IKK phosphorylation was observed. These results collectively indicate that the PMC-mediated inhibition of NF-κB activity in LPS/IFN-γ-stimulated VSMCs occurs through the ROS-PP2A-p65 signalling cascade, an IKK-IκBα-independent mechanism. Therapeutic interventions using PMC may therefore be beneficial for the treatment of vascular inflammatory diseases.

## Introduction

Experimental and clinical studies have indicated the role of oxidative stress induced by reactive oxygen and nitrogen species in the pathology of various diseases, including cancer, ageing and cardiovascular disease [1,2], thus drawing attention to the therapeutic use of antioxidants. Vitamin E is the major lipid soluble antioxidant of lipoproteins and biological membranes, and exists as tocopherol and tocotrienol forms in mammalian tissues [3]. However, clinical trials have not revealed strong evidence to support the use of vitamin E for the prevention or treatment of such diseases [4,5], and the results of a meta-analysis even indicated that a vitamin E supplement greater than 400 U/day could increase all-cause mortality among adult patients with chronic diseases [6].

**Fig. 1:**
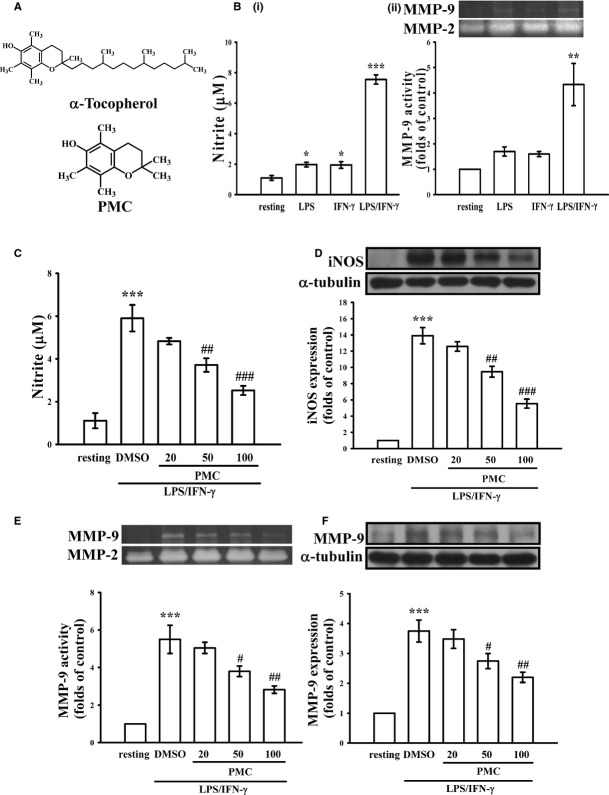
(**A**) Chemical structures of α-tocopherol and 2,2,5,7,8-pentamethyl-6-hydroxychromane (PMC), (**B**–**E**) the effects of PMC on expression of iNOS and MMP-9 in LPS/IFN-γ-stimulated VSMCs. (**B**) VSMCs were treated with PBS (resting group), LPS (50 μg/ml), IFN-γ (100 U/ml) or a mixture of LPS (50 μg/ml) and IFN-γ (100 U/ml) for 24 hrs. (i) Nitrite concentration and (ii) MMP-9 activity were evaluated as described in the Materials and methods. (**C**–**E**) VSMCs were treated with PBS (resting group) or pre-treated with PMC (20–100 μM) or an equal volume of DMSO (solvent control) for 20 min., followed by the addition of a mixture of LPS (50 μg/ml) and IFN-γ (100 U/ml) for 24 hrs. (**C**) Nitrite concentration, (**D**) iNOS protein level, (**E**) MMP-9 activity and (**F**) MMP-9 protein level were evaluated as described in the Materials and methods. *P < 0.05, **P < 0.01 and ***P < 0.001, compared with the resting group. ^#^*P* < 0.05, ^##^*P* < 0.01 and ^###^*P* < 0.001, compared with the LPS/IFN-γ group. The data are presented as the mean ± SEM (*n* = 3).

The α-tocopherol derivative, 2,2,5,7,8-pentamethyl-6-hydroxychromane (PMC), is more hydrophilic than other vitamin E derivatives (Fig. 1A), and has been shown to have potent free radical-scavenging activities and low cytotoxicity [7]. Treatment with PMC inhibits platelet aggregation and improves neurological function in ischaemia-reperfusion brain injury-related disorders [8,9]. We recently reported that PMC suppresses platelet-derived growth factor (PDGF)-BB-stimulated vascular smooth muscle cell (VSMC) proliferation *in vitro* and reduces balloon injury-induced neointimal formation *in vivo* [10]. Consequently, PMC represents a promising alternative to vitamin E in clinical applications.

The vascular inflammatory response involves complex interactions among immunomodulatory cells, endothelial cells and VSMCs. Persistent increases in inflammatory cytokines derived from immune cells, endothelial cells and VSMCs have been implicated in vascular dysfunction and vascular diseases, such as atherosclerosis, abdominal aortic aneurysms and hypertension [11,12]. Cytokines, such as tumour necrosis factors, interleukins and interferons (IFNs), interact with specific receptors and activate signalling cascades, leading to various inflammatory responses involving matrix metalloproteinase (MMP) expression, the production of nitric oxide and reactive oxygen species (ROS), cell growth, cell adhesion and cell migration [11,12]. In addition, increasing evidence from animal studies suggests that pattern-recognition receptors, such as Toll-like receptor 4, mediate various cardiovascular inflammatory diseases, including sepsis, septic shock and atherosclerosis [13].

Pharmacological approaches used to diminish the effects of cytokines and pathogens may provide new strategies for managing these inflammatory vascular diseases. Previous studies have demonstrated that inducible nitric oxide synthase (iNOS) expression is increased in VSMCs after exposure to lipopolysaccharide (LPS) or cytokines [14], and that the effect of LPS combined with one or more cytokines is an additive [15]. We examined the mechanisms underlying the inhibitory effects of PMC on the expression of iNOS and MMP-9 in rat VSMCs co-stimulated by LPS and IFN-γ to investigate the potentially protective effects of PMC treatment in vascular inflammatory conditions.

## Materials and methods

### Animals and reagents

Male Wistar rats were purchased from BioLASCO (Taipei, Taiwan). DMEM, trypsin (0.25%), L-glutamine, penicillin/streptomycin and foetal bovine serum (FBS) were purchased from Invitrogen (Life Technologies, Carlsbad, CA, USA). Dimethyl sulphoxide (DMSO), LPS, PMC and 2,7-dichlorofluorescin diacetate (DCF-DA) were purchased from Sigma-Aldrich (St. Louis, MO, USA). The recombinant rat IFN-γ was purchased from PeproTech (Rocky Hill, NJ, USA).

The anti-iNOS rabbit polyclonal antibody (pAb), the anti-p65, anti-phospho-protein phosphatase 2A (PP2A) and anti-demethyl-PP2A mouse monoclonal antibodies (mAbs), and the protein A/G plus agarose beads were purchased from Santa Cruz Biotechnology (Dallas, TX, USA). The anti-α-tubulin and anti-MMP-9 mouse mAb were purchased from Thermo Scientific (Waltham, MA, USA). The anti-phospho-p65 (Ser^536^) rabbit pAb was purchased from Cell Signaling (Danvers, MA, USA). The anti-IκB kinase (IKK; Ser^180^/Ser^181^) rabbit pAb was purchased from Assay Biotechnology (Sunnyvale, CA, USA). The PP2A-c rabbit pAb was purchased from GeneTex (Irvine, CA, USA). The anti-phospho-ribosomal s6 kinase (RSK)-1 rabbit mAb was purchased from Epitomics (Burlingame, CA, USA).

The Hybond-P polyvinylidene difluoride (PVDF) membrane, the enhanced chemiluminescence (ECL) Western blotting detection reagent and analysis system, the horseradish peroxidase (HRP)-conjugated donkey anti-rabbit IgG pAb, and the sheep antimouse IgG pAb were purchased from GE Healthcare Life Sciences (Waukesha, WI, USA). The reporter plasmid expressing firefly luciferase under the control of the nuclear factor (NF)-κB promoter was constructed by inserting four tandem copies of the NF-κB-promoter sequence into the pTAL-Luc plasmid (Clontech Laboratories, Mountain View, CA, USA). The pRL-TK Renilla luciferase vector (Promega, Madison, WI, USA) was cotransfected to serve as an internal control ensure transfection efficiency. The Dual-Glo Luciferase Assay System was also purchased from Promega. PMC was dissolved in DMSO and stored at 4°C until used.

### VSMC isolation and culture

All procedures involving animals were performed according to the Guide for the Care and Use of Laboratory Animals (National Academy Press, Washington, DC, 1996). The thoracic aorta was removed from the male Wistar rats (250–300 g), and the vessels were stripped of the endothelium and adventitia. The VSMCs were obtained by combined collagenase and elastase digestion [16] of the stripped aorta. The VSMCs were grown in DMEM supplemented with 20 mM HEPES, 10% FBS, 1% penicillin/streptomycin and 2 mM L-glutamine at 37°C in a humidified atmosphere of 5% CO_2_. The VSMCs from passages 4 to 8 were used in all the experiments. The primary cultured aortic VSMCs showed the ‘hills and valleys’ pattern, and the expression of α-smooth muscle actin was confirmed (data not showed).

### Determination of nitrite concentration

To determine nitric oxide production, the level of nitrite, a stable oxidative end product of nitric oxide, was measured using a previously described colorimetric method [14] with minor modifications. The VSMCs (5 × 10^5^ cells/dish) were seeded in DMEM containing 10% FBS in 6-cm culture dishes, and grown until the monolayer was confluent. The cells were pre-treated with PMC (20–100 μM) or an equal volume of solvent control (0.1% DMSO) for 20 min. The cells were treated with a mixture of 50 μg/ml LPS and 100 U/ml IFN-γ (LPS/IFN-γ) or with an equal volume of the solvent control for 24 hrs. The media were collected for nitrite analysis. Nitrite accumulation was determined using the Griess reagent (1% sulphanilamide and 0.1% naphthalenediamine in 2.5% phosphoric acid). The absorbance at 550 nm was measured using an MRX microplate reader (Dynex, Chantilly, VA, USA). Nitrite concentrations were extrapolated from a standard curve constructed using linear regression of the absorbance measurements of sodium nitrite standard solutions prepared using the same culture medium.

### Immunoblotting study

Immunoblotting analysis was used to examine protein expression in VSMCs as described previously [10]. The VSMCs (5 × 10^5^ cells/dish) were seeded in DMEM containing 10% FBS in 6-cm culture dishes, and grown until the monolayer was confluent. The cells were pre-treated with PMC (20–100 μM) or an equal volume of solvent control (0.1% DMSO) for 20 min. The cells were treated with LPS/IFN-γ, 1 mM H_2_O_2_ or an equal volume of the solvent control for the indicated times as experimental design, after which the cellular proteins were extracted with lysis buffer. Aliquots of 50 μg of soluble protein were subjected to SDS-PAGE, and the resolved protein bands were electrophoretically transferred onto 0.45 μm PVDF membranes. The membranes were blocked with TBST (10 mM Tris-base, 100 mM NaCl and 0.01% Tween 20) containing 5% skimmed milk for 1 hr and probed with the various primary antibodies before incubation with HRP-linked antimouse IgG or anti-rabbit IgG (diluted 1: 3000 in TBST) for 1 hr. Immunoreactive bands were detected using an ECL system. Protein expression was quantified by videodensitometry using the Biolight Windows Application, V2000.01 (Bio-Profil, Vilber Lourmat, France).

### Gelatin zymography

The cultured media harvested from cells were analysed for proteins based on gelatinolytic activity using gelatin zymography as described previously [17]. The VSMCs (5 × 10^5^ cells/dish) were seeded in DMEM containing 10% FBS in 6-cm culture dishes, and grown until the monolayer was confluent. The cells were pre-treated with PMC (20–100 μM) or an equal volume of solvent control (0.1% DMSO) for 20 min., and then treated with LPS/IFN-γ or an equal volume of solvent control for 24 hrs. Conditioned media were resuspended in a non-reducing sample buffer, and subjected to SDS-PAGE on a 10% acrylamide gel copolymerized with 1% gelatin. After electrophoresis, the gels were washed with 2.5% Triton X-100 for 1 hr and incubated in an enzyme buffer (50 mM Tris-HCl, pH 7.5, 20 mM NaCl, 5 mM CaCl_2_ and 0.02% Brij-35) at 37°C for 72 hrs. The gels were stained with 0.5% Coomassie Brilliant Blue G-250 (Bio-Rad, Hercules, CA, USA). Following destaining in 25% methanol and 10% acetic acid, gelatinolytic proteins were visualized as clear bands against a blue-stained background. Gels were scanned, and densitometric analysis was performed with the Quantity One (Bio-Rad) image analysis program. Molecular weights corresponding to the protein bands were estimated by comparison with pre-stained molecular-weight protein markers.

### Separation of cytoplasmic and nuclear extracts

The cytoplasmic and nuclear protein fractions were separated as described previously [17]. Cells were lysed in a hypotonic buffer (10 mM KCl, 0.5 mM DTT, 10 mM aprotinin, 10 mM leupeptin, 20 mM PMSF and 10 mM HEPES, pH 7.9) for 15 min. on ice. After vortexing for 10 sec., the nuclei were pelleted by centrifugation at 15,000 × g for 1 min., the supernatants (cytoplasmic extract) were immediately transferred to a clean pre-chilled tube and put on ice. The pelleted nuclei were resuspended in a hypertonic buffer (25% glycerol, 1.5 mM MgCl_2_, 4 mM EDTA, 0.05 mM DTT, 10 mM aprotinin, 10 mM leupeptin, 20 mM PMSF and 20 mM HEPES, pH 7.6) for 30 min. on ice. The proteins in the supernatants (nuclear extract) were collected by centrifugation at 15,000 × *g* for 2 min. then immediately transferred to a clean pre-chilled tube and put on ice. These protein extracts were stored until use.

### Immunofluorescence confocal laser scanning microscopy

A confocal microscopic analysis was performed to identify and evaluate the translocation of p65 in VSMCs. Vascular smooth muscle cells (1 × 10^5^ cells/cover slide) were placed on cover slides and allowed to adhere in a cell culture incubator overnight. Vascular smooth muscle cells were treated as the design of the experiment, then fixed with 4% paraformaldehyde for 30 min. and permeabilized with 80% methanol for 15 min. After incubation with 3% skimmed milk in PBS for 60 min., the preparation was incubated for 1 hr with a primary Ab (anti-p65 mouse mAb; 1:80). Cells were then washed three times with PBS and exposed to the secondary Ab [FITC-conjugated antimouse immunoglobin G (IgG; Santa Cruz Biotechnology) at 1:100, 1% BSA/PBS] for 60 min. Slides were counterstained with 0.1 μg/ml DAPI (Sigma-Aldrich) and mounted with mounting buffer (Vector Laboratories, Burlingame, CA, USA) under a glass coverslip on a Leica TCS SP5 Confocal Spectral Microscope Imaging System using an argon/krypton laser (Mannheim, Germany).

### Transfection and NF-κB-luciferase assays

The VSMCs (2 × 10^5^ cells/well) were seeded in DMEM containing 10% FBS in 12-well plates, and grown until the monolayer was 60–70% confluent. The cells were transfected with a mixture of the NF-κB-luc and Renilla-luc plasmids by using the Lipofectamine reagent (Invitrogen) according to the manufacturer's instructions. The transfected VSMCs were pre-treated with PMC (20–100 μM) or an equal volume of solvent control (0.1% DMSO) for 20 min., followed by treatment with LPS/IFN-γ or an equal volume of solvent control for 24 hrs. After the cells were harvested, the luciferase activity was quantified using the Dual-Glo Luciferase Assay System (Promega), and was normalized based on Renilla luciferase activity. The level of induction was calculated as the ratio of the normalized luciferase activity of LPS/IFN-γ stimulated cells to that of non-stimulated cells.

### Suppression of PP2A Expression

The suppression of *pp2a* gene expression was performed with a previously described method [17]. Pre-designed siRNAs targeting the mouse PP2A mRNA sequence, the negative control siRNA and the 3′dT overhangs were purchased from Ambion (Austin, TX, USA). The sequence of the siRNA oligonucleotide targeting the coding region of the PP2A catalytic subunit (PP2A-c) mRNA was 5′-CCAUACUCCGAGGGAAUCATT-3′. The negative control siRNA consisted of a 19-bp scrambled sequence.

### Immunoprecipitation assays

Immunoprecipitation (IP) was used to demonstrate the interaction between p65 and PP2A-c as described previously [18]. The VSMCs (5 × 10^5^ cells/dish) were seeded in DMEM containing 10% FBS in 6-cm culture dishes, and grown until the monolayer was confluent. The cells were stimulated with LPS/IFN-γ for 15–60 min. and lysed in an IP buffer. An equal amount of protein from each supernatant was pre-cleared by rotation with protein A/G-agarose-conjugated beads for 2 hrs. Samples were rotated overnight with 1 mg/ml anti-PP2A-c mAb. The following day, 20 μl of A/G-agarose-conjugated beads were added, and the mixture was rotated overnight. The immunoprecipitates were washed three times with an IP buffer, and analysed by immunoblotting.

### Measurement of intracellular ROS

The VSMCs (5 × 10^5^ cells/dish) were seeded in DMEM containing 10% FBS in 6-cm culture dishes, and grown until the monolayer was confluent. The cells were treated with DCF-DA (20 μM) for 20 min. After treatment with PMC (20 and 50 μM) or an equal volume of solvent control (0.1% DMSO) for 20 min., cells were stimulated with PDGF-BB (10 ng/ml or LPS/IFN-γ. The cells were washed with PBS before trypsinization. The levels of intracellular ROS were detected using a flow cytometry instrument (Beckman Coulter, Brea, CA, USA). Data were collected from 10,000 cells per experimental group. All experiments were repeated at least three times to ensure reproducibility.

### Statistical analysis

The experimental results are expressed as the mean ± standard error, and are accompanied by the number of observations (*n*) from different biological replicates. The data were assessed using anova. If anova indicated significant differences among group means, an additional comparison was performed with the Newman–Keuls method. A *P* < 0.05 was considered to indicate a statistically significant difference.

## Results

### Effects of PMC on the expressions of iNOS and MMP-9 in LPS/IFN-γ-stimulated VSMCs

As shown in Figure 1B, the synergistic effects of LPS (50 μg/ml) and IFN-γ (100 U/ml) on the production of nitric oxide and expression of MMP-9 were occurred in VSMCs. We thus used the combination of LPS and IFN-γ (LPS/IFN-γ) in all of our experiments. In Figure 1C, the treatment of VSMCs with LPS/IFN-γ for 24 hrs increased nitric oxide production from 1.1 ± 0.4 to 5.9 ± 0.6 μM (*P* < 0.001; *n* = 3), and the pre-treatment of PMC (20, 50, and 100 μM) inhibited nitric oxide production in a concentration-dependent manner in LPS/IFN-γ-stimulated VSMCs by 22.9%, 45.8% and 70.8% respectively. We next examined whether the protein level of iNOS, which catalyses nitric oxide formation, is affected by PMC in LPS/IFN-γ-stimulated VSMCs. As shown in Figure 1D, treatment with LPS/IFN-γ increased iNOS expression 13.9 ± 1.0-fold, compared with the control group (*P* < 0.001, *n* = 3). Concentration-dependent inhibition was observed in LPS/IFN-γ stimulated VSMCs in response to treatment with PMC (20, 50 and 100 μM), with decreases to 10.1%, 34.1% and 65.1% respectively. These results suggest that the PMC-mediated inhibition of LPS/IFN-γ-induced nitric oxide formation resulted from the down-regulation of iNOS expression.

Under vascular inflammatory conditions, cytokines can increase the production and processing of MMPs from inactive zymogens to active enzymes in VSMCs [19]. We investigated whether PMC regulates the expression of MMP-2 and MMP-9 in LPS/IFN-γ-treated VSMCs. As shown in Figure 1E, LPS/IFN-γ treatment increased MMP-9 activation 5.5 ± 0.8-fold compared with the control group (*P* < 0.001, *n* = 3), and pre-treatment with PMC (20, 50 and 100 μM) inhibited MMP-9 activity in LPS/IFN-γ-stimulated VSMCs, with decreases to 11.1%, 37.8% and 60.0% respectively. In contrast, PMC had no effect on MMP-2 activity in the absence or presence of LPS/IFN-γ (Fig. 1E). Immunoblotting analysis shown in Figure 1F further demonstrates that treatment with PMC (50 and 100 μM) reduced the level of MMP-9 proteins in LPS/IFN-γ-stimulated VSMCs to 37.0% and 55.6% respectively. These findings collectively indicate that PMC inhibits LPS/IFN-γ-induced vascular inflammatory responses in VSMCs, including decreased iNOS expression, nitric oxide formation and MMP-9 expression.

### Effects of PMC on the NF-κB-signalling pathway in LPS/IFN-γ-stimulated VSMCs

**Fig. 2:**
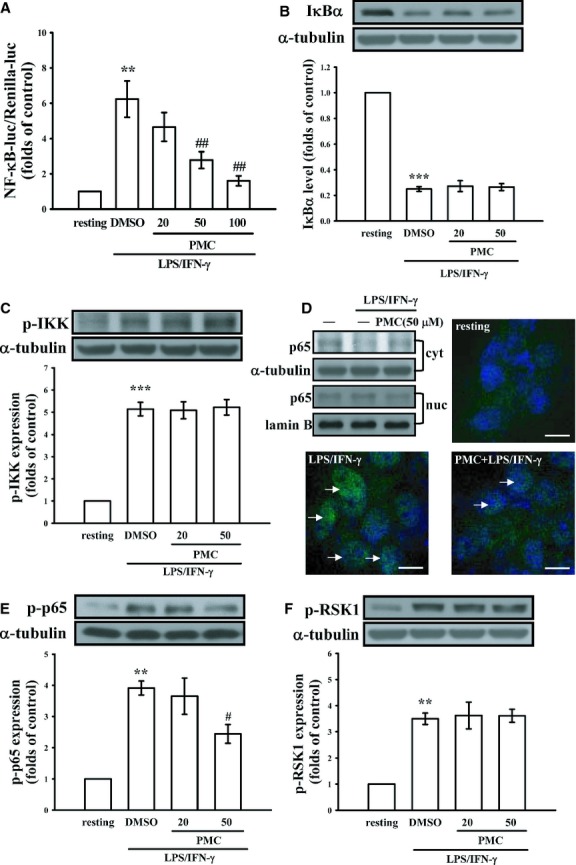
Effects of PMC on the NF-κB-signalling pathway in LPS/IFN-γ-stimulated VSMCs. (**A**) VSMCs were transiently transfected with NF-κB luc and Renilla luc for 24 hrs. After transfection, cells were treated with PBS (resting group) or pre-treated with PMC (20–100 μM) or an equal volume of DMSO (solvent control) for 20 min., followed by the addition of a mixture of LPS (50 μg/ml) and IFN-γ (100 U/ml) for 24 hrs. To determine the effects of PMC on NF-κB-related signalling molecules, VSMCs were treated with PBS (resting group) or pre-treated with PMC (20 and 50 μM) or an equal volume of DMSO (solvent control) for 20 min., followed by the addition of a mixture of LPS (50 μg/ml) and IFN-γ (100 U/ml) for 30 min. (**B**) IκBα degradation, (**C**) IKK phosphorylation, (**D**) translocation of p65, (**E**) p65 phosphorylation and (**F**) RSK-1 phosphorylation were determined using an immunoblotting assay and confocal microscopic analysis as described in the Materials and methods. **P < 0.01 and ***P < 0.001, compared with the resting group. ^#^*P* < 0.05 and ^##^*P* < 0.01, compared with the LPS/IFN-γ group. The data are presented as the mean ± SEM (*n* = 3). Confocal images are typical of those obtained in three separate experiments demonstrating the distribution of p65 (arrows) in VSMCs. Blue depicts the nucleus and green depicts p65 protein. The white bar indicates 10 μm.

To clarify the inhibitory mechanism of PMC on the expression of iNOS and MMP-9, we examined the promoter activity of NF-κB, a transcription factor that regulates the expression of many inflammatory proteins, including MMP-9 and iNOS [20,21]. A reporter assay was used to determine the effect of PMC on the transactivation of the NF-κB promoter in LPS/IFN-γ-stimulated VSMCs. As shown in Figure 2A, the VSMCs treated with LPS/IFN-γ for 24 hrs exhibited a 6.2 ± 1.0-fold increase in NF-κB-driven luciferase expression, compared with the control group (*P* < 0.01; *n* = 3). The LPS/IFN-γ-induced increase in NF-κB-luciferase activity was markedly suppressed in VSMCs pre-treated with 50 or 100 μM PMC to 65.4% and 88.4% respectively (*n* = 3; Fig. 2A).

We next evaluated the effect of PMC on IκBα and critical upstream signalling molecules, including IκB kinases α and β (IKKα and IKKβ respectively) [22], in LPS/IFN-γ-stimulated VSMCs. However, the PMC pre-treatment did not reverse IκBα degradation or suppress IKKs phosphorylation (Fig. 2B and C respectively). We then examined whether PMC interferes with NF-κB subcellular localization. The NF-κB/p65 complex has been known to shuttle from the cytoplasm to the nucleus following various inflammatory stimuli [23]. As shown in Figure 2D, no matter by immunoblotting or confocal microscopic assay, p65 exhibited a decreased cytoplasmic localization and increased nuclear localization following LPS/IFN-γ treatment, and this phenomenon was markedly restored following treatment with 50 μM PMC.

Several studies have shown the phosphorylation of p65 at critical serine residues may mediate its dimerization, DNA binding and nuclear localization [24]. As shown in Figure 2E, LPS/IFN-γ-induced p65 phosphorylation in VSMCs pre-treated with 50 μM PMC was significantly inhibited (51.7%), compared with p65 phosphorylation in LPS/IFN-γ-stimulated VSMCs without PMC (*P* < 0.05, *n* = 3; Fig. 2E). These results suggest that p65 phosphorylation may be responsible for the PMC-mediated inhibition of NF-κB transactivation in LPS/IFN-γ-stimulated VSMCs.

We next investigated the possible mechanism underlying the PMC-mediated suppression of p65 phosphorylation. We examined the phosphorylation of RSK1, an IKK-independent regulator of p65 phosphorylation [25]. However, PMC had no effect on RSK1 phosphorylation in LPS/IFN-γ-stimulated VSMCs (Fig. 2F).

### Role of PP2A in the inhibitory effects of PMC in LPS/IFN-γ-stimulated VSMCs

**Fig. 3:**
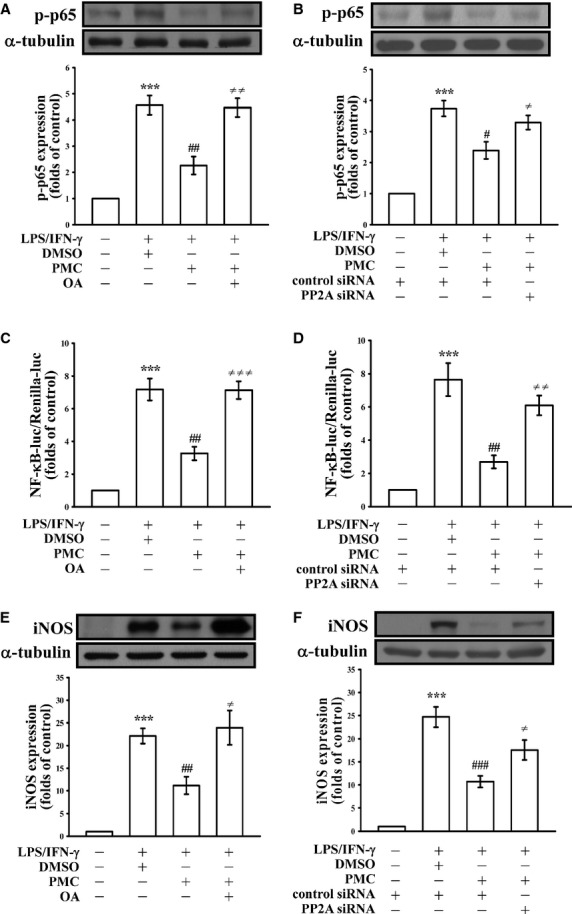
Role of PP2A in the inhibitory effects of PMC in LPS/IFN-γ-stimulated VSMCs. (**A**, **C**, and **E**) VSMCs were pre-treated with 10 nM okadaic acid (OA) or transiently transfected with scrambled siRNA (control) or PP2A siRNA (**B**, **D**, and **F**), and then treated with PMC (50 μM), followed by the addition of a mixture of LPS (50 μg/ml) and IFN-γ (100 U/ml). (**A** and **B**) p65 phosphorylation (**C** and **D**) NF-κB transactivation and (**E** and **F**) iNOS expression were determined as described in the Materials and methods. ***P < 0.001, compared with the control group; ^#^*P* < 0.05, and ^##^*P* < 0.01, compared with the LPS/IFN-γ group. ^≠^*P* < 0.05, ^≠≠^*P* < 0.01 and ^≠≠≠^*P* < 0.001, compared with the OA group (**A**, **C**, and **E**) or PP2A siRNA group (**B**, **D**, and **F**). The data are presented as the mean ± SEM (*n* = 3).

The regulation of the phosphorylated protein is balanced by the activities of kinases and phosphatases [26]. We thus examined whether PP2A, a predominant protein phosphatase in mammalian cells [27], is involved in the PMC-mediated p65 dephosphorylation in LPS/IFN-γ-stimulated VSMCs. As shown in Figure 3A, pre-treatment with 10 nM okadaic acid (OA), a PP2A selective inhibitor, significantly restored PMC-mediated inhibition of p65 phosphorylation in LPS/IFN-γ-stimulated VSMCs. In addition, the PMC-mediated down-regulation of NF-κB-driven luciferase activity in LPS/IFN-γ-stimulated VSMCs was also reversed by pre-treatment with OA (Fig. 3C).

To confirm the effects of PMC in LPS/IFN-γ-stimulated VSMCs are mediated by PP2A, we transfected VSMCs with PP2A siRNA, and found the inhibitory effect of PMC on p65 phosphorylation and NF-κB-luciferase activity was significantly reversed by knockdown of the *pp2a* gene (Fig. 3B and D). Pre-treatment with OA and transfection of VSMCs with PP2A siRNA also significantly reversed the PMC-mediated reduction in iNOS expression in LPS/IFN-γ-stimulated VSMCs (Fig. 3E and F respectively). These data suggest that PP2A plays an important role in the inhibitory effects of PMC in LPS/IFN-γ-activated VSMCs.

**Fig. 4:**
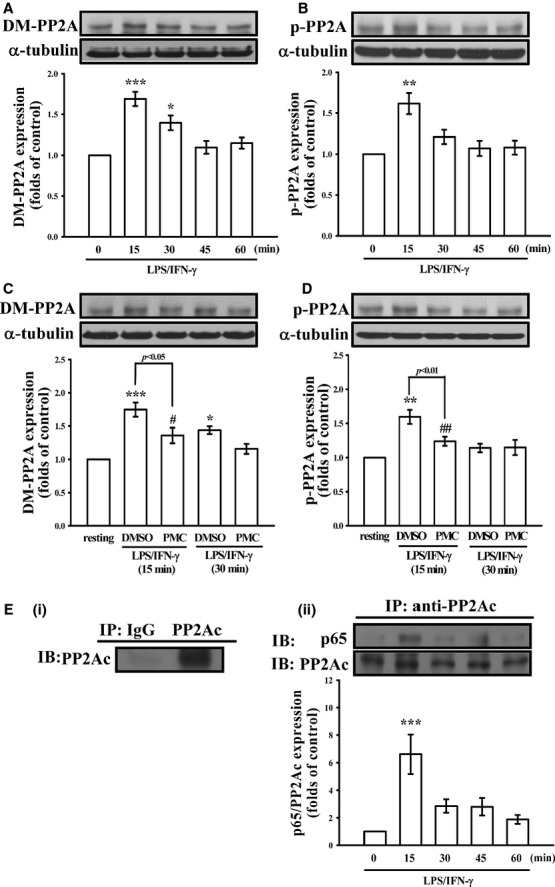
Effects of PMC on the regulation of PP2A in LPS/IFN-γ-stimulated VSMCs. (**A**–**D**) The VSMCs were treated with PBS (resting group) or pre-treated with 50 μM PMC or an equal volume of DMSO (solvent control) for 20 min., followed by the addition of a mixture of LPS (50 μg/ml) and IFN-γ (100 U/ml) for the indicated periods. The demethylation and phosphorylation of PP2A were analysed by immunoblotting assay using anti-phospho-protein phosphatase 2A(p-PP2A) and anti-demethyl-PP2A(DM-PP2A) antibodies (**E**). (i) The cell extracts of VSMCs were immunoprecipitated by control rabbit IgG or anti-PP2A-c antibody, and the precipitated proteins were analysed by immunoblotting with an anti-PP2A-c antibody. (ii) The VSMCs were stimulated with LPS/IFN-γ for 0–60 min. The cell extracts were immunoprecipitated using an anti-PP2A-c antibody, and the precipitated proteins were analysed by immunoblotting with an anti-p65 antibody. *P < 0.05, **P < 0.01 and ***P < 0.001, compared with the control group. ^#^*P* < 0.05 and ^##^*P* < 0.01, compared with the LPS/IFN-γ group. The data are presented as the mean ± SEM (*n* = 3).

We further evaluated the effects of PMC on PP2A activation in LPS/IFN-γ-stimulated VSMCs. As shown in Figure 4A and B, demethylation and phosphorylation of PP2A significantly increased in VSMCs following treatment with LPS/IFN-γ for 15 min. Pre-treatment with 50 μM PMC significantly reduced PP2A demethylation and phosphorylation in LPS/IFN-γ-stimulated VSMCs (Fig. 4C and D). In addition, we examined the colocalization of PP2A and p65 by IP with an anti-PP2A-c antibody, followed by immunoblotting with an anti-p65 antibody. As shown in Figure 4E, p65 was immunoprecipitated with PP2A-c after treatment with LPS/IFN-γ for 15 min., indicating that p65 interacted directly with PP2A-c after LPS/IFN-γ induction before dissociating gradually.

### Involvement of ROS in the inhibitory effects of PMC in VSMCs stimulated by LPS/IFN-γ

**Fig. 5:**
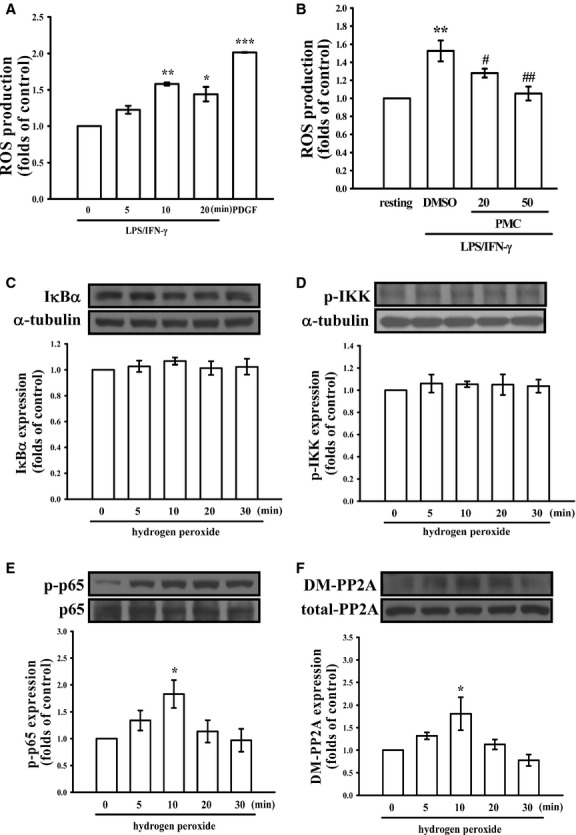
Role of ROS in the inhibitory effects of PMC in LPS/IFN-γ-stimulated VSMCs. (**A** and **B**) The VSMCs were treated with PBS (resting group) or pre-treated with PMC (20 or 50 μM) or an equal volume of DMSO (solvent control) for 20 min., followed by the addition of a mixture of LPS (50 μg/ml) and IFN-γ (100 U/ml) for the indicated periods or the addition of platelet-derived growth factor-BB (10 ng/ml) for 10 min. to trigger ROS formation. (**C**–**F**) The VSMCs were stimulated with 1 mM H_2_O_2_ for 0–60 min. to trigger IκBα degradation, IKK and p65 phosphorylation, and PP2A demethylation. *P < 0.05, **P < 0.01 and ***P < 0.001, compared with the control group. ^#^*P* < 0.05 and ^##^*P* < 0.01, compared with the LPS/IFN-γ group. The data are presented as the mean ± SEM (*n* = 3).

To determine the mechanism underlying the PMC-mediated regulation of PP2A activation in LPS/IFN-γ-stimulated VSMCs, we examined whether PMC-induced PP2A activation occurs through the antioxidant properties of PMC. Figure 5A shows that treatment with LPS/IFN-γ for 10 min. significantly increased ROS production (1.6 ± 0.0-fold) in VSMCs, compared with the control group (*P* < 0.01, *n* = 3). Treatment with 20 or 50 μM PMC inhibited ROS production in LPS/IFN-γ-stimulated VSMCs to 47.1% and 90.1% respectively (Fig. 5B). In addition, IκBα degradation and IKK phosphorylation were not significantly altered in LPS/IFN-γ-stimulated VSMCs treated with H_2_O_2_ (Fig. 5C and D), whereas p65 phosphorylation and PP2A demethylation increased markedly (Fig. 5E and F).

## Discussion

Randomized clinical trials have failed to reveal the efficacy of antioxidants, particularly that of vitamin E, for the clinical application [4,5]. Previous strategies to relieve oxidative stress in patients aimed to improve antioxidant levels in target tissues by increasing the cellular intake. However, a pro-oxidant effect of high-dose vitamin E supplementation may cause the increase in mortality observed in intervention studies using this nutrient [28]. Among vitamin E analogues, PMC has been shown to have the most potent anti-oxidative effect on lipid peroxidation, and the least pro-oxidant influence on hepatocytes [7]. Our previous studies have shown that PMC provides potent anti-thrombotic, anti-ischaemic and anti-atherosclerotic effects [8–10], and exhibited lower cytotoxicity in VSMCs, compared with vitamin E [10]. In our present study, PMC significantly suppressed the expression of iNOS and MMP-9 in LPS/IFN-γ-stimulated VSMCs, indicating that PMC may be a potential alternative for vitamin E for the prevention and treatment of vascular inflammatory diseases.

The activation of NF-κB is tightly controlled to ensure a functional host defence and prevent hyper-inflammation and tumourigenesis [22]. The most common form of NF-κB in most cell types is the p65/p50 heterodimer, and NF-κB signalling is governed by the IKK complex, which consists of IKKα, IKK β, IKKγ and the downstream substrate IκBα. After stimulation, activated IKK phosphorylates IκBα, leading to IκBα degradation, enhanced NF-κB nuclear translocation, and subsequent transcriptional activation [29]. However, neither IκBα degradation nor IKK was affected by PMC in this study. In addition to IκB, the phosphorylation of the NF-κB subunit p65 represents another mechanism regulating NF-κB nuclear trafficking and transcriptional activity [22]. In this study, we found that the inhibition of p65 phosphorylation may be related to the PMC-mediated inhibition of NF-κB in LPS/IFN-γ-stimulated VSMCs. In the previous study, RSK1 was shown to regulate p65 phosphorylation and NF-κB activation *via* an IKK-independent pathway [25]. Nevertheless, PMC had no effect on the phosphorylation of RSK1 in VSMCs.

Activation of an unknown protein phosphatase is thus postulated to be required for the attenuation of PMC on NF-κB activity by inhibiting p65 phosphorylation. We found that both treatment with OA, and the knockdown of *pp2a* in LPS/IFN-γ-stimulated VSMCs reversed the PMC-mediated inhibition of the NF-κB-promoter activity, p65 phosphorylation and iNOS expression, which is consistent with the results of a previous study that showed that PP2A regulates the activation of NF-κB [30]. Thus, our results indicate that PP2A is involved in the PMC-mediated inhibition of NF-κB activity and p65 phosphorylation, and plays a pivotal role in the inhibitory effects of PMC in an IKK-independent manner.

The PP2A family of enzymes represents a major class of serine, threonine protein phosphatases, which play important roles in the development, cell death and regulation of numerous signalling pathways. Accounting for up to 1% of total cellular protein and 80% of total serine/threonine phosphatases, PP2A represents a major proportion of protein phosphatases in mammalian cells [31]. The core enzyme of PP2A consists of a scaffold subunit (the A subunit) and a catalytic subunit (the C subunit) that interact with a variable regulatory B subunit to comprise the holoenzyme [31], which undergoes various post-translational modifications that regulate the activation of PP2A.

The reversible methylation of the PP2A core enzyme is a conserved regulatory mechanism, and the methylation of L309 in a conserved TPDYFL motif in the C terminus of PP2A-c has been shown to enhance holoenzyme assembly and phosphatase activity [31]. In addition to reversible methylation, PP2A-c has been shown to undergo tyrosine phosphorylation at Y307. However, in contrast to methylation, tyrosine phosphorylation has been found to inhibit the catalytic function of PP2A [32]. In the present study, the LPS/IFN-γ-induced demethylation and phosphorylation of PP2A was reversed in PMC-treated VSMCs. In addition, the IP data revealed that PP2A-c colocalizes with p65 through a direct interaction (Fig. 4E). This finding is consistent with a previous study showing that PP2A interacts with and directly dephosphorylates RelA in melanoma cells, and they also found OA could restore p65 dephosphorylation [33]. These evidence further support our finding that PMC inhibits p65 phosphorylation in LPS/IFN-γ-stimulated VSMCs through the regulation of PP2A.

**Fig. 6:**
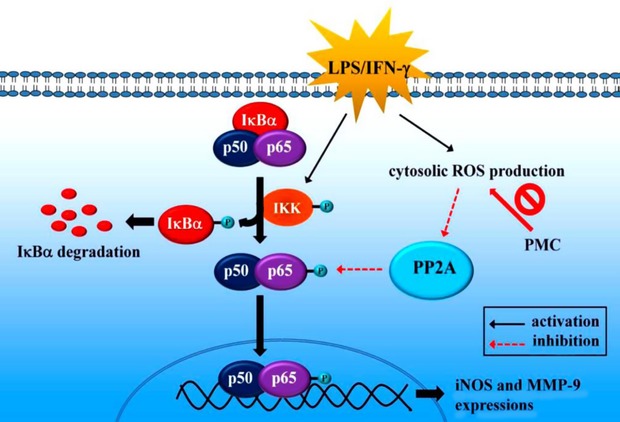
Diagram of the hypothetical inhibitory mechanism of PMC-induced effects in LPS/IFN-γ-stimulated VSMCs. The LPS combined with IFN-γ trigger the expression of inflammatory proteins, including iNOS and MMP-9, through IκBα- (IKK-IκBα-p65) and IκBα-independent (ROS-PP2A-p65) pathways.

To clarify the precise mechanism by which PMC regulates PP2A activity and suppresses NF-κB signalling, we examined the role of ROS in the PMC-mediated regulation of PP2A and the NF-κB-signalling pathway. Our findings revealed PMC could inhibit LPS/IFN-γ-induced ROS production in VSMCs. In addition, p65 phosphorylation and PP2A inactivation was induced, but neither IκBα degradation nor IKK phosphorylation was observed in H_2_O_2_-treated VSMCs. These data imply that the ROS-PP2A-p65 signalling cascade is involved in the inhibitory effects of PMC in LPS/IFN-γ-stimulated VSMCs (Fig. 6), which is consistent with the findings of previous studies that indicated that ROS inactivate PP2A, leading to the activation of downstream signalling pathways [34,35].

The regulatory mechanisms underlying the effect of ROS on NF-κB activation largely remain unclear. A high degree of complexity characterizes ROS interactions with NF-κB pathways because different ROS can exhibit different effects at different sites simultaneously, and the effects of many ROS are cell-type specific [36]. Our current study provides the first evidence that ROS regulates NF-κB activation in LPS/IFN-γ-stimulated VSMCs through the inactivation of PP2A-mediated p65 dephosphorylation, and suggests a possible mechanism to clarify the IKK-IκBα-independent inhibition of NF-κB activation by another antioxidant, N-acetylcysteine (NAC) [37]. Indeed, we have found that the treatment of NAC could inhibit p65 phosphorylation and PP2A demethylation in LPS/IFN-γ-activated VSMCs, and the IκBα-independent inhibition of NF-κB activation by PMC also occurred in TNF-α-stimulated VSMCs (data not shown). These data collectively indicated that this ROS-mediated IκBα-independent regulation on NF-κB activation may not be restricted by the specificity of stimulators or ROS scavengers.

Multiple lines of evidence suggest that the nitric oxide-derived oxidant, peroxynitrite, contributes to inflammatory cardiovascular diseases, such as atherogenesis [38]. In addition, MMPs also play important roles in vascular remodelling and subsequent pathological events in the progression of diabetes, coronary arterial diseases and atherosclerosis [39]. Our findings show that PMC significantly diminished the expression of iNOS and MMP-9 in LPS/IFN-γ-stimulated VSMCs through the attenuation of the ROS-PP2A-p65 signalling cascade, and our data also suggest that the IKK-IκBα-independent inhibition of NF-κB activation may occur through the regulation of PP2A-induced inhibition of p65 phosphorylation. In conclusion, PMC may represent a superior alternative to vitamin E as a potential therapeutic agent for the treatment and prevention of inflammatory vascular diseases.

## Supporting information

**Figure S1** The role of NADPH oxidase in LPS/IFN-γ-induced ROS production in VSMCs. **Figure S2** Effects of PMC on IκBα degradation and p65 phosphorylation in TNF-α-activated VSMCs.

**Figure S3** Effects of PMC on iNOS expression in H_2_O_2_-activated VSMCs. **Figure S4** Evaluation of PP2A activity in H_2_O_2_-treated vascular smooth muscle cells (VSMCs).

**Figure S5** Effects of N-acetylcysteine (NAC) on p65 phosphorylation and dimethyl-PP2A (DM-PP2A) expression in LPS/IFN-γ-activated VSMCs.
