# Supplementary figures and images for: PMC, a potent hydrophilic α-tocopherol derivative, inhibits NF-κB activation via PP2A but not IκBα-dependent signals in vascular smooth muscle cells
Source: J Cell Mol Med. 2014 Apr 13;18(7):1278–89. doi: 10.1111/jcmm.12277 (PMC4124013; doi:10.1111/jcmm.12277)

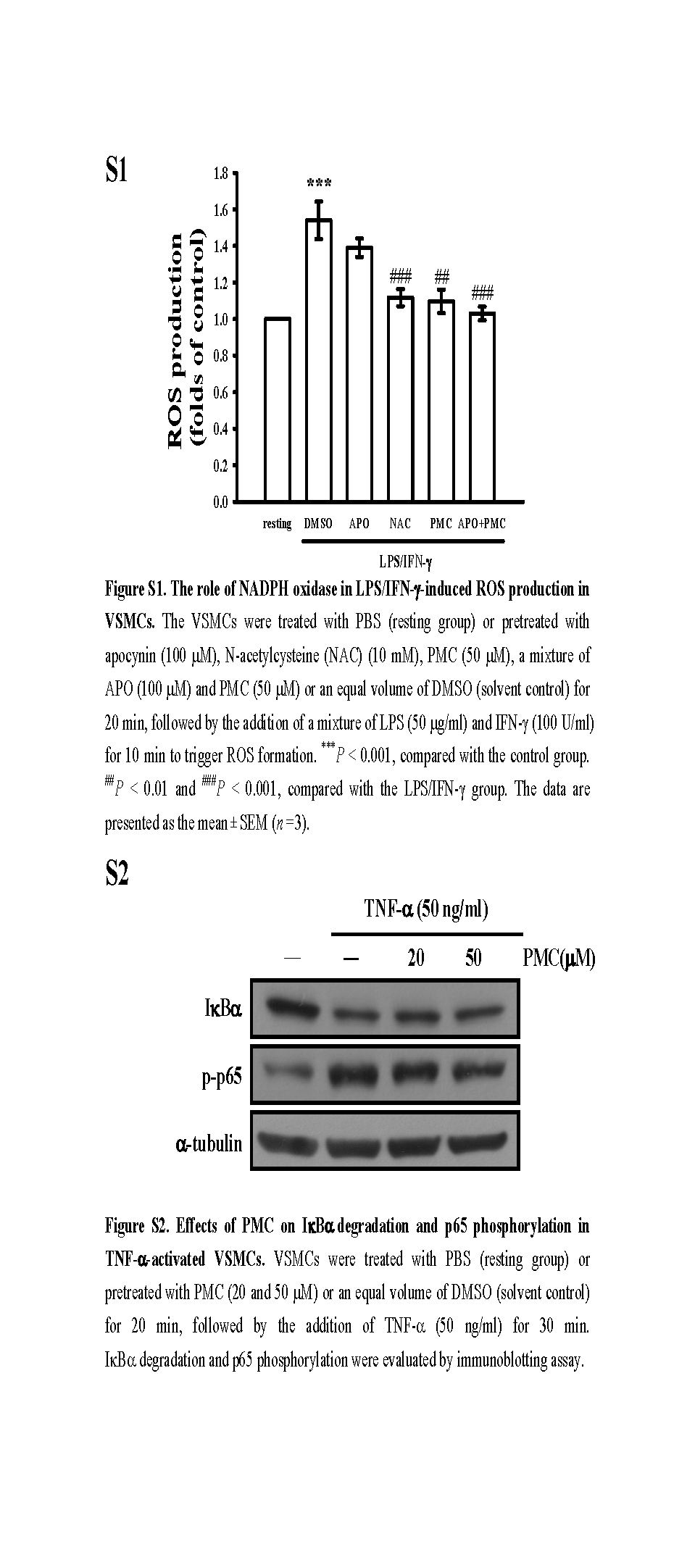

Supplement: Supplementary file 1 [file jcmm0018-1278-SD1.tiff]

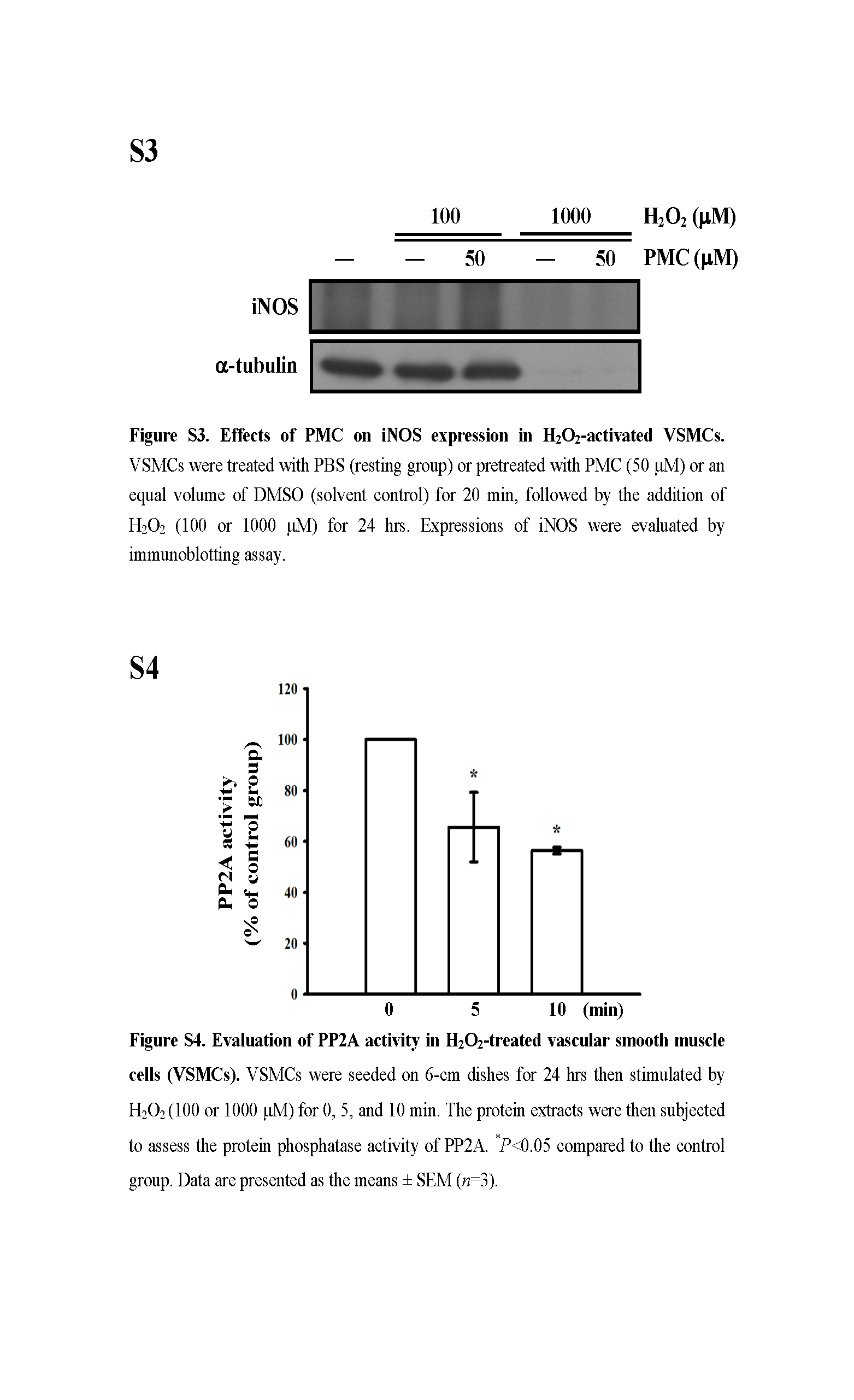

Supplement: Supplementary file 2 [file jcmm0018-1278-SD2.tiff]

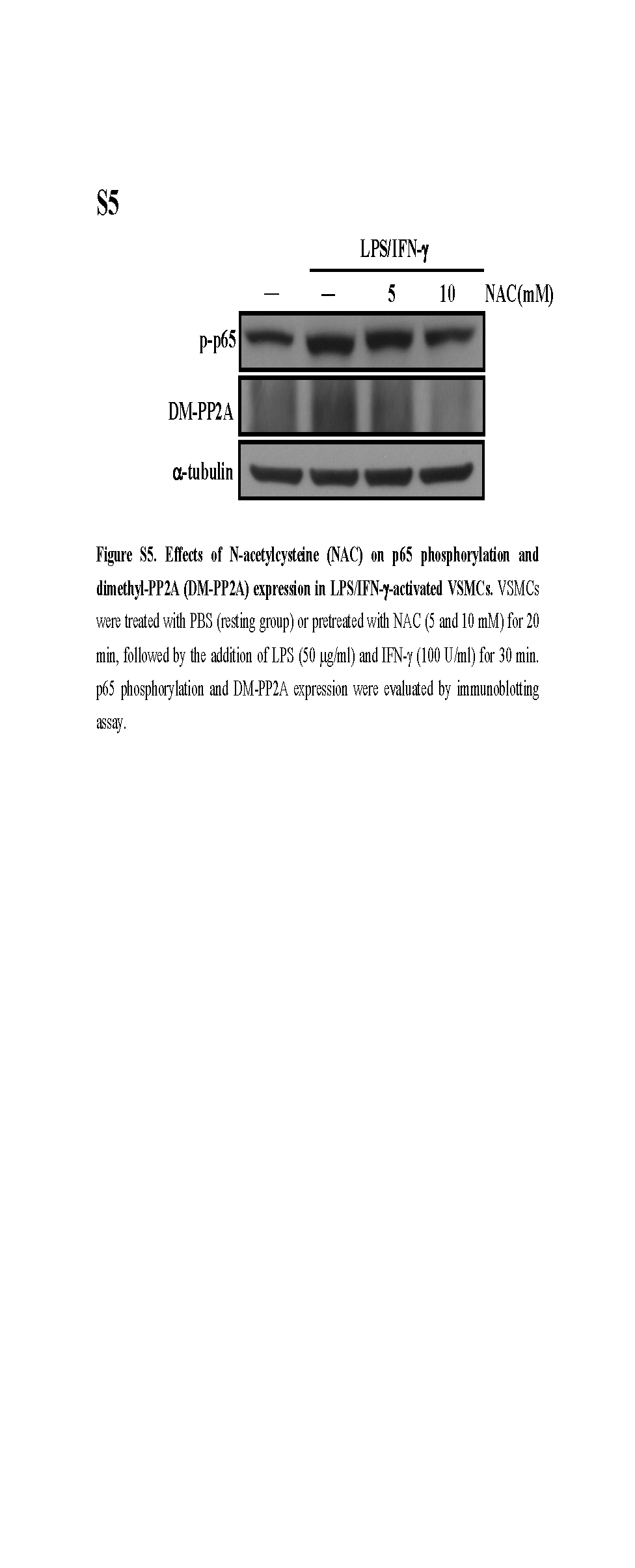

Supplement: Supplementary file 3 — Figure S5 Effects of N-acetylcysteine (NAC) on p65 phosphorylation and dimethyl-PP2A (DM-PP2A) expression in LPS/IFN-γ-activated VSMCs. [file jcmm0018-1278-SD3.tiff]
